# Point-of-care Ultrasound Diagnosis of Slipped Capital Femoral Epiphysis

**DOI:** 10.5811/cpcem.2019.1.41357

**Published:** 2019-01-22

**Authors:** Imran Asad, Michelle Sin Lee

**Affiliations:** University of Toronto, Hospital for Sick Children, Division of Pediatric Emergency Medicine, Department of Pediatrics, Toronto, Canada

## CASE PRESENTATION

**Image 1:**
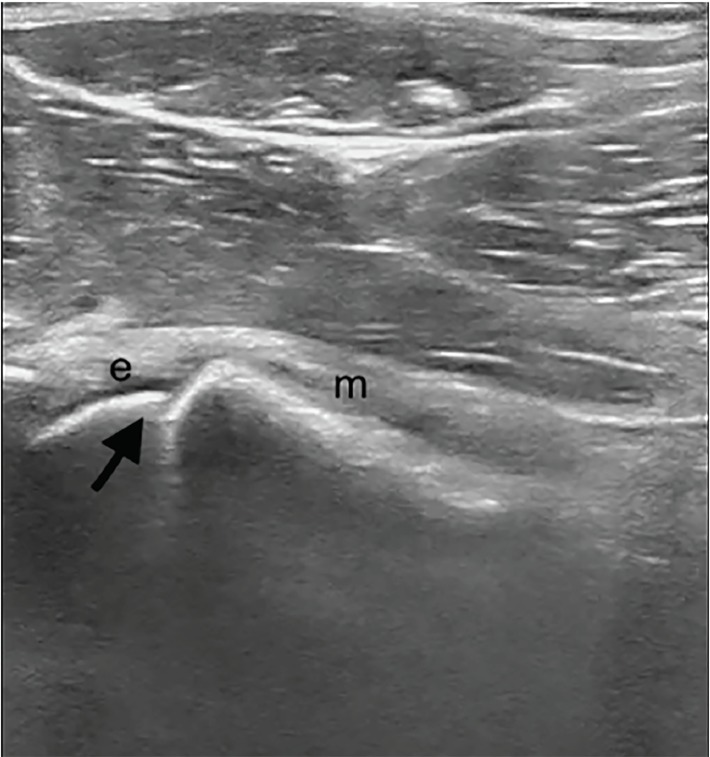
Point-of-care ultrasound image of left hip showing displacement of epiphysis(e) from metaphysis (m) – the physeal step (arrow).

**Image 2:**
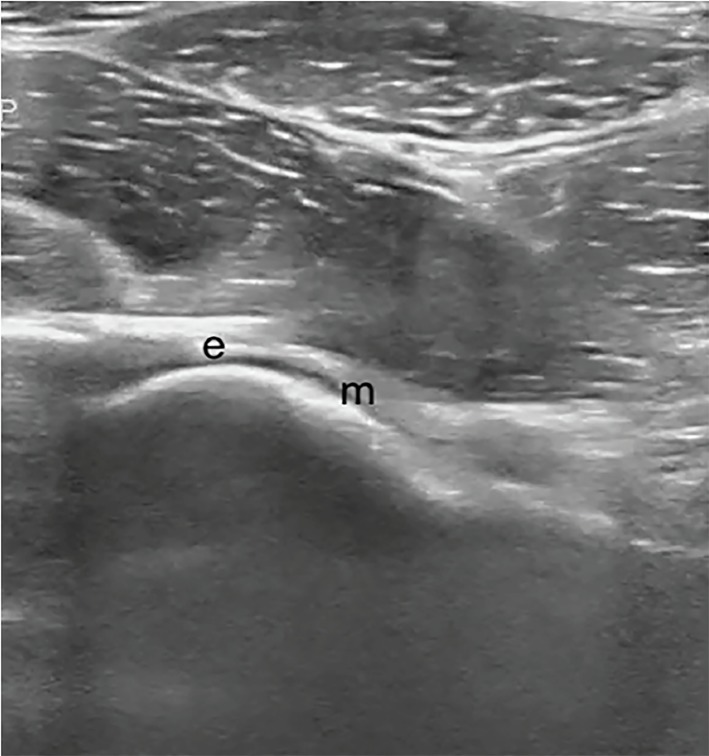
Point-of-care ultrasound image of right hip showing normal contour of metaphysis (m) and epiphysis (e) with no displacement.

**Image 3:**
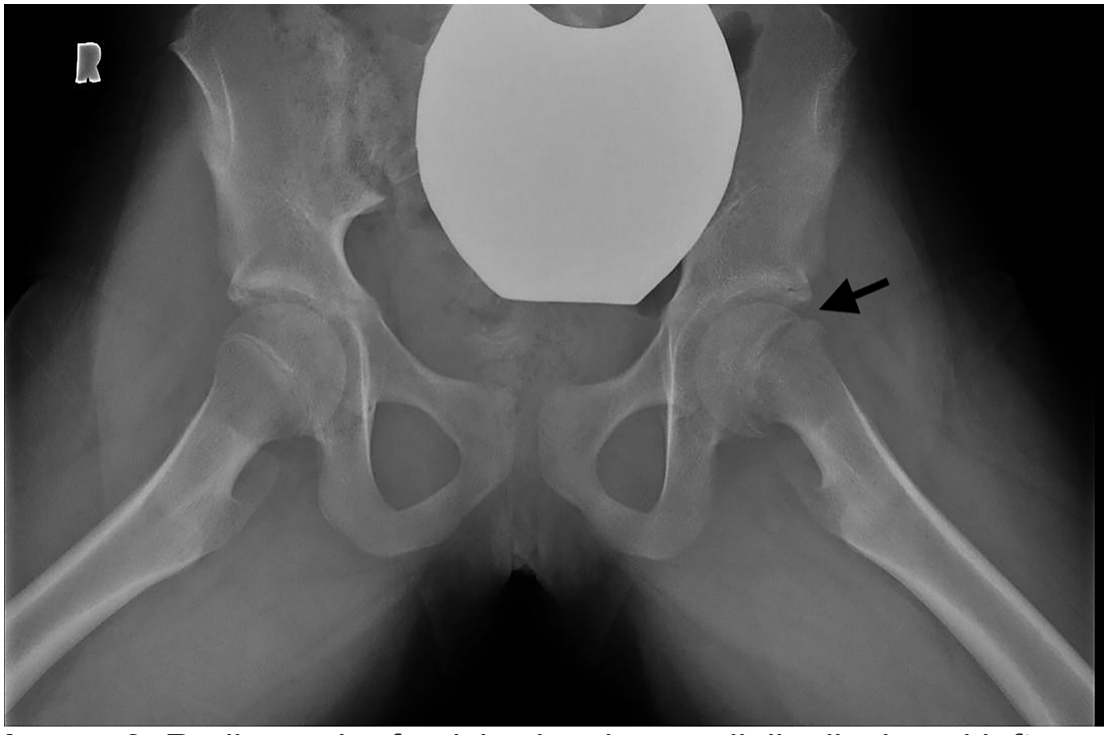
Radiograph of pelvis showing medially displaced left femoral epiphysis (arrow).

An 11-year-old female was brought to the emergency department with left hip and knee pain as well as limping for three weeks. There was no fever or recent trauma. Physical examination revealed restricted range of movement due to pain on hip flexion, internal and external rotation. A point-of-care ultrasound (POCUS) performed by an emergency physician (Image 1) raised the suspicion for her diagnosis when compared with right side (Image 2), which prompted expedited immobilization and pain control. POCUS was performed using a linear, high-frequency probe (14–5 MegaHertz) aligned parallel to the femoral neck. Subsequently, her pelvic radiograph (Image 3) confirmed the diagnosis.

## DISCUSSION

Slipped capital femoral epiphysis (SCFE) is an important hip disorder of adolescence commonly occurring between the ages of 8–15 years. SCFE is characterized by a displacement of the capital femoral epiphysis from the metaphysis (femoral neck), through the growth plate.^1^ SCFE usually presents with sudden or progressive limping with hip, groin, thigh or even knee pain.^1^ Delayed diagnosis has been associated with increased severity of slip and complications, including avascular necrosis of the femoral head, chondrolysis and osteoarthritis.^2^

Although plain radiographs are the primary modality used to diagnose SCFE, ultrasound has also been used for diagnosis, staging and follow-up management of SCFE.^3^ Key ultrasound findings include posterior displacement of epiphysis with a physeal step, reduced distance between the anterior rim of the acetabulum and the metaphysis, remodeling of the metaphysis and, occasionally, joint effusion.^4^ Ultrasound sensitivity in diagnosis of SCFE is as high as 95%^3^, Its point-of-care use by emergency physicians can be a useful adjunct as a non-radiating, readily available bedside modality for assessing the limping child – especially in low-resource or rural settings where radiography may not be readily available or would require subsequent transfer to a different facility.

The patient underwent open reduction and internal fixation with uneventful recovery.

**CPC-EM Capsule**

What do we already know about this clinical entity?

*Slipped capital femoral epiphysis (SCFE) is a disorder of older children and adolescents presenting with progressive unilateral pain and limp*.

What is the major impact of the image(s)?

*These images demonstrate sonographic findings of SCFE, particularly epiphyseal displacement from metaphysis of femur when compared with unaffected side*.

How might this improve emergency medicine practice?

*Point-of-care ultrasound provides a rapid, non-ionizing bedside method to diagnose SCFE, allowing early immobilization, pain control and expedited management in the emergency department*.
